# 2,4,6,8-Tetra­kis(2-methoxy­phen­yl)-3,7-diaza­bicyclo­[3.3.1]nonan-9-one diethyl ether hemisolvate

**DOI:** 10.1107/S1600536809036733

**Published:** 2009-09-19

**Authors:** Hoong-Kun Fun, Chin Sing Yeap, K. Rajesh, S. Sarveswari, V. Vijayakumar

**Affiliations:** a X-ray Crystallography Unit, School of Physics, Universiti Sains Malaysia, 11800 USM, Penang, Malaysia; b Organic Chemistry Division, School of Science and Humanities, VIT University, Vellore 632 014, India

## Abstract

In the title compound, C_35_H_36_N_2_O_5_·0.5C_4_H_10_O, the asymmetric unit contains one bicyclo­[3.3.1]nonane mol­ecule and a half-occupancy diethyl ether solvent with the O atom lying on a crystallographic inversion center. Two intra­molecular N—H⋯O hydrogen bonds generate *S*(6) ring motifs. The bicyclo­[3.3.1]nonane ring system adopts a chair-boat conformation. In the crystal structure, the mol­ecules are linked by weak inter­molecular C—H⋯N hydro­gen bonds into chains along the *b* axis; additional stabilization is provide by C—H⋯π inter­actions.

## Related literature

For applications of bicyclo­[3.3.1]nonane derivatives, see: Arias-Perez *et al.* (1997 ▶). For applications of *N*,*N*-diphenyl derivatives, see: Srikrishna & Vijayakumar (1998 ▶). For bicyclic systems with aryl groups, see: Vijayakumar *et al.* (2000 ▶). For ring conformations, see: Cremer & Pople (1975 ▶). For hydrogen-bond motifs, see: Bernstein *et al.* (1995 ▶). For the stability of the temperature controller used for the data collection, see: Cosier & Glazer (1986 ▶).
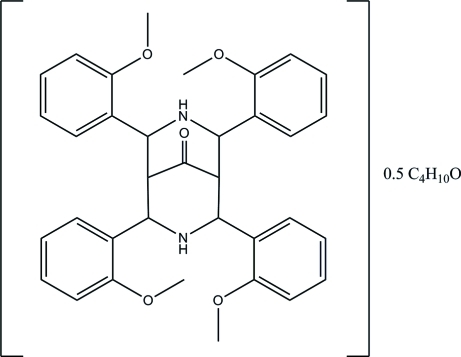


## Experimental

### 

#### Crystal data

C_35_H_36_N_2_O_5_·0.5C_4_H_10_O
*M* *_r_* = 1203.44
Monoclinic, 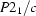

*a* = 13.5607 (2) Å
*b* = 13.7640 (2) Å
*c* = 20.3227 (3) Å
β = 123.143 (1)°
*V* = 3176.10 (8) Å^3^
*Z* = 2
Mo *K*α radiation
μ = 0.08 mm^−1^
*T* = 100 K
0.36 × 0.26 × 0.24 mm

#### Data collection

Bruker SMART APEXII CCD area-detector diffractometer
Absorption correction: multi-scan (**SADABS**; Bruker, 2005 ▶) *T*_min_ = 0.971, *T*_max_ = 0.980
49818 measured reflections
11431 independent reflections
8831 reflections with *I* > 2σ(*I*)
*R*_int_ = 0.035

#### Refinement

*R*[*F* ^2^ > 2σ(*F* ^2^)] = 0.054
*wR*(*F* ^2^) = 0.144
*S* = 1.03
11431 reflections
426 parameters
H-atom parameters constrained
Δρ_max_ = 0.58 e Å^−3^
Δρ_min_ = −0.43 e Å^−3^

### 

Data collection: *APEX2* (Bruker, 2005 ▶); cell refinement: *SAINT* (Bruker, 2005 ▶); data reduction: *SAINT*; program(s) used to solve structure: *SHELXTL* (Sheldrick, 2008 ▶); program(s) used to refine structure: *SHELXTL*; molecular graphics: *SHELXTL*; software used to prepare material for publication: *SHELXTL* and *PLATON* (Spek, 2009 ▶).

## Supplementary Material

Crystal structure: contains datablocks global, I. DOI: 10.1107/S1600536809036733/lh2903sup1.cif

Structure factors: contains datablocks I. DOI: 10.1107/S1600536809036733/lh2903Isup2.hkl

Additional supplementary materials:  crystallographic information; 3D view; checkCIF report

## Figures and Tables

**Table 1:** Hydrogen-bond geometry (Å, °)

*D*—H⋯*A*	*D*—H	H⋯*A*	*D*⋯*A*	*D*—H⋯*A*
N2—H2⋯O4	0.87	2.19	2.8184 (13)	129
N2—H2⋯O5	0.87	2.32	2.8936 (15)	124
C4—H4*A*⋯N2^i^	0.93	2.60	3.470 (2)	156
N1—H1⋯*Cg*1^ii^	0.87	2.66	3.4167 (10)	146
C34—H34*C*⋯*Cg*2^iii^	0.96	2.70	3.5099 (16)	143

Symmetry codes: (i) 
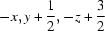
; (ii) 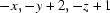; (iii) 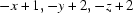. *Cg*1 and *Cg*2 are the centroids of the C12–C17 and C19–C24 benzene rings, respectively.

**Fig. 1.**
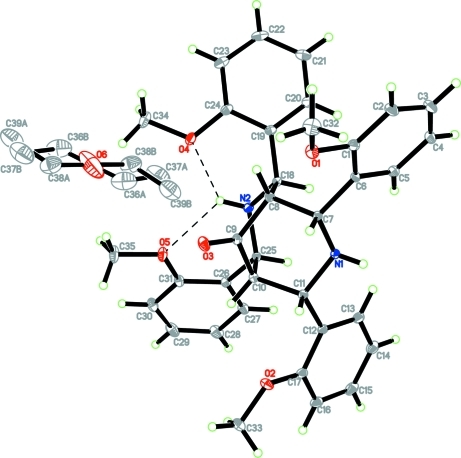
The molecular structure of the title compound with atom labels and 30% probability ellipsoids for non-H atoms. Atoms labelled with suffix B are generated by symmetry code (-x + 1, -y + 1, -z + 2). Intramolecular hydrogen bonds are shown in as dashed lines. Hydrogen atoms of the solvent molecules have been omittted for clarity.

**Fig. 2.**
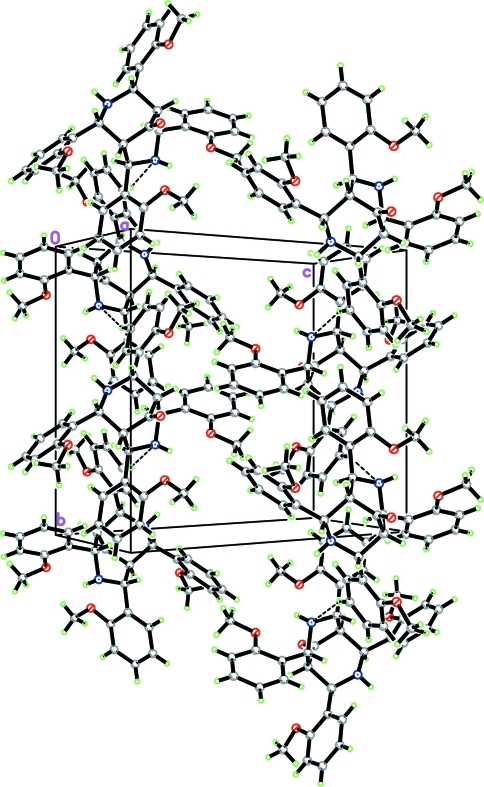
The crystal packing of title compound, showing the molecules linked along the b axis. The solvent molecules are omitted for clarity. Intermolecular hydrogen bonds are shown as dashed lines.

## supplementary crystallographic information

### Comment

Bicyclo[3.3.1]nonane moieties are present in many biologically active molecules like alkaloids and drugs (Arias-Perez *el al.*, 1997). Functionalized 3-azabicyclo[3.3.1]nonanes have been studied intensively because of their pharmaceutical use and these compounds find applications as an important class of organic compounds in the field of molecular recognition. The 1,5-diphenyl-3,7-diazabicyclo[3.3.1]nonan-9-ones are local anaesthetics. Some of them have hypotensive activity. *N*,*N*-diphenyl derivatives are found to possess antiphogistic and anti-thrombic activities (Srikrishna & Vijayakumar, 1998). The synthesis and stereochemistry of 3,7-diazabicyclo[3.3.1]nonan-9-ones and their derivatives are of much interest due to their diverse biological activities, such as antibacterial, antifungal, antiarrhythmic, antiphologistic, antithrombic, calcium antagonistic, hypotensive and neuroleptic properties and also because of their presence in naturally occurring lupin alkaloids. The conformational analysis of 3,7-diazabicyclo[3.3.1]nonanes (bispidines) is of considerable interest both from the theoretical view point and due to their biological activity. In recent years the 2,4,6,8-tetraaryl-3,7-diazabicyclo[3.3.1]nonanes constitutes an interesting case for study because of the presence of four aryl groups. If all the aryls are in equatorial orientations, molecular models indicate close proximity of the aryls in both rings in the bicyclic systems (Vijayakumar *el al.*, 2000). If they are in the twin chair conformation, it causes severe non-bonded interactions between aryl groups in 2,8-positions and 4,6-positions. So in order to attain the stability, the system may exist in the twin chair conformations and the aryls may assume different orientations in order that the overall stability can be attained. Hence these systems constitute an interesting case for study.

In the title compound (Fig. 1), the asymmetric unit contains one bicyclo[3.3.1]nonane molecule and a half-occupied diethyl ether solvent with the oxygen atom of diethyl ether molecule lying on the crystallographic inversion center (1/2, 1/2, 0). The intramolecular N2—H2···O4 and N2—H2···O5 hydrogen bonds form six-membered rings with *S*(6) ring motifs (Bernstein *et al.*, 1995). The bicyclo[3.3.1]nonane ring adopts a chair-boat conformation with puckering parameter Q = 0.6509 (12) Å, θ = 8.67 (11)°, φ = 131.5 (7)° for one of the piperidine rings (N1/C7–C11) and Q = 0.7318 (12) Å, θ = 90.93 (9)°, φ = 6.12 (10)° for the other piperidine ring (N2/C18/C8–C10/C25) (Cremer & Pople, 1975). The N atoms adopt a pyramidal configuration. The phenyl rings substituted at C7 (C1–C6) and C11 (C12–C17) positions are oriented with one another with an angle of 23.02 (6)° whereas the phenyl rings that substituted at C18 (C19–C24) and C25 (C26–C31) are oriented with one another at an angle of 55.08 (7)°. Two methoxyphenyl groups substituted at C7 and C11 are in equatorial orientations with torsion angles C6–C7–C8–C9 = -171.93 (9)° and C9–C10–C11–C12 = -175.76 (9)°. The other two methoxyphenyl groups substituted at C18 and C25 have torsion angles of C9–C8–C18–C19 = 132.02 (9)° and C9–C10–C25–C26 = -120.19 (9)°.

In the crystal structure, the molecules are linked by intermolecular C4—H4A···N2 hydongen bonds into one-dimensional chains along the *b* axis. The molecules are also stabilized by the C—H···π interactions.

### Experimental

A mixture of acetone (0.68 ml), 2-methoxybenzaldehyde (5 g) and dry ammonium acetate (1.4 g) was taken in 1:4:2 molar ratio in ethanol and it were heated on water bath till the colour changes to reddish orange. The mixture was allowed to stand for 24 h resulting in the formation of a sticky substance. To that diethyl ether was added and warmed gently. The fine needle-shaped crystals were separated out from the reaction mixture upon slow evaporation of the solvent. The purity of the compound was checked by TLC. Yield: 53%. *M.p.* 507 K.

### Refinement

N-bound hydrogen atoms were located from the difference Fourier map and refined riding on their parent atom with *U*_iso_(H) = 1.2*U*_eq_(N). The rest of the hydrogen atoms were positioned geometrically [C–H = 0.9300–0.9800 Å] and refined using a riding model, with *U*_iso_(H) = 1.2*U*_eq_(C) and 1.5*U*_eq_(methyl C). A rotating group model was used for the methyl groups excepting for those in the solvent molecule.

### Crystal data

**Table:**

C_35_H_36_N_2_O_5_·0.5C_4_H_10_O	*F*(000) = 1284
*M**_r_* = 1203.44	*D*_x_ = 1.258 Mg m^−^^3^
Monoclinic, *P*2_1_/*c*	Mo *K*α radiation, λ = 0.71073 Å
Hall symbol: -P 2ybc	Cell parameters from 9998 reflections
*a* = 13.5607 (2) Å	θ = 2.3–32.3°
*b* = 13.7640 (2) Å	µ = 0.08 mm^−^^1^
*c* = 20.3227 (3) Å	*T* = 100 K
β = 123.143 (1)°	Block, colourless
*V* = 3176.10 (8) Å^3^	0.36 × 0.26 × 0.24 mm
*Z* = 2	

### Data collection

**Table:**

Bruker SMART APEXII CCD area-detector diffractometer	11431 independent reflections
Radiation source: fine-focus sealed tube	8831 reflections with *I* > 2σ(*I*)
graphite	*R*_int_ = 0.035
φ and ω scans	θ_max_ = 32.5°, θ_min_ = 2.1°
Absorption correction: multi-scan (*SADABS*; Bruker, 2005)	*h* = −20→19
*T*_min_ = 0.971, *T*_max_ = 0.980	*k* = −20→18
49818 measured reflections	*l* = −28→30

### Refinement

**Table:**

Refinement on *F*^2^	Primary atom site location: structure-invariant direct methods
Least-squares matrix: full	Secondary atom site location: difference Fourier map
*R*[*F*^2^ > 2σ(*F*^2^)] = 0.054	Hydrogen site location: inferred from neighbouring sites
*wR*(*F*^2^) = 0.144	H-atom parameters constrained
*S* = 1.03	*w* = 1/[σ^2^(*F*_o_^2^) + (0.0696*P*)^2^ + 1.0838*P*] where *P* = (*F*_o_^2^ + 2*F*_c_^2^)/3
11431 reflections	(Δ/σ)_max_ = 0.001
426 parameters	Δρ_max_ = 0.58 e Å^−^^3^
0 restraints	Δρ_min_ = −0.43 e Å^−^^3^

### Special details

**Table:**

Experimental. The crystal was placed in the cold stream of an Oxford Cyrosystems Cobra open-flow nitrogen cryostat (Cosier & Glazer, 1986) operating at 100.0 (1) K.
Geometry. All e.s.d.'s (except the e.s.d. in the dihedral angle between two l.s. planes) are estimated using the full covariance matrix. The cell e.s.d.'s are taken into account individually in the estimation of e.s.d.'s in distances, angles and torsion angles; correlations between e.s.d.'s in cell parameters are only used when they are defined by crystal symmetry. An approximate (isotropic) treatment of cell e.s.d.'s is used for estimating e.s.d.'s involving l.s. planes.
Refinement. Refinement of *F*^2^ against ALL reflections. The weighted *R*-factor *wR* and goodness of fit *S* are based on *F*^2^, conventional *R*-factors *R* are based on *F*, with *F* set to zero for negative *F*^2^. The threshold expression of *F*^2^ > σ(*F*^2^) is used only for calculating *R*-factors(gt) *etc*. and is not relevant to the choice of reflections for refinement. *R*-factors based on *F*^2^ are statistically about twice as large as those based on *F*, and *R*- factors based on ALL data will be even larger.

### Fractional atomic coordinates and isotropic or equivalent isotropic displacement parameters (Å^2^)

**Table:**

	*x*	*y*	*z*	*U*_iso_*/*U*_eq_	Occ. (<1)
O1	0.37715 (7)	1.20002 (6)	0.79917 (5)	0.02045 (17)	
O2	0.16165 (8)	0.81583 (7)	0.50351 (5)	0.02487 (19)	
O3	0.44172 (7)	0.91411 (6)	0.75132 (5)	0.02168 (18)	
O4	0.44782 (7)	0.85185 (6)	0.93266 (5)	0.02092 (18)	
O5	0.39565 (7)	0.69167 (6)	0.78054 (5)	0.02172 (18)	
N1	0.12334 (8)	1.00157 (7)	0.64982 (5)	0.01379 (17)	
H1	0.0770	1.0444	0.6148	0.017*	
N2	0.23043 (8)	0.81126 (6)	0.79232 (5)	0.01415 (17)	
H2	0.3021	0.7893	0.8231	0.017*	
C1	0.26664 (10)	1.21183 (8)	0.78518 (6)	0.0165 (2)	
C2	0.23225 (12)	1.29360 (9)	0.80816 (7)	0.0238 (2)	
H2A	0.2848	1.3445	0.8341	0.029*	
C3	0.11833 (13)	1.29837 (10)	0.79187 (8)	0.0272 (3)	
H3A	0.0948	1.3530	0.8068	0.033*	
C4	0.03991 (11)	1.22298 (10)	0.75379 (7)	0.0237 (2)	
H4A	−0.0357	1.2264	0.7437	0.028*	
C5	0.07496 (10)	1.14171 (9)	0.73055 (6)	0.0182 (2)	
H5A	0.0221	1.0909	0.7050	0.022*	
C6	0.18748 (9)	1.13516 (8)	0.74484 (6)	0.01415 (19)	
C7	0.22560 (9)	1.05217 (7)	0.71488 (6)	0.01278 (18)	
H7A	0.2718	1.0791	0.6953	0.015*	
C8	0.30324 (9)	0.97559 (7)	0.77992 (6)	0.01276 (18)	
H8A	0.3710	1.0073	0.8254	0.015*	
C9	0.34324 (9)	0.91109 (8)	0.73914 (6)	0.01396 (19)	
C10	0.24244 (9)	0.85293 (7)	0.67446 (6)	0.01329 (18)	
H10A	0.2718	0.8096	0.6506	0.016*	
C11	0.15541 (9)	0.92867 (8)	0.61200 (6)	0.01379 (19)	
H11A	0.1944	0.9612	0.5893	0.017*	
C12	0.04571 (10)	0.87765 (8)	0.54689 (6)	0.0154 (2)	
C13	−0.06243 (10)	0.88483 (9)	0.53918 (7)	0.0187 (2)	
H13A	−0.0697	0.9256	0.5728	0.022*	
C14	−0.16013 (11)	0.83219 (9)	0.48217 (7)	0.0246 (3)	
H14A	−0.2320	0.8382	0.4775	0.029*	
C15	−0.14919 (12)	0.77071 (9)	0.43244 (7)	0.0272 (3)	
H15A	−0.2138	0.7348	0.3948	0.033*	
C16	−0.04261 (12)	0.76229 (9)	0.43826 (7)	0.0248 (3)	
H16A	−0.0359	0.7208	0.4048	0.030*	
C17	0.05428 (11)	0.81632 (8)	0.49459 (6)	0.0190 (2)	
C18	0.23146 (9)	0.91662 (7)	0.80614 (6)	0.01316 (18)	
H18A	0.1497	0.9390	0.7736	0.016*	
C19	0.27336 (9)	0.93751 (8)	0.89074 (6)	0.01536 (19)	
C20	0.20440 (11)	0.99246 (9)	0.90835 (7)	0.0198 (2)	
H20A	0.1319	1.0158	0.8675	0.024*	
C21	0.24159 (12)	1.01339 (10)	0.98597 (7)	0.0256 (3)	
H21A	0.1946	1.0505	0.9966	0.031*	
C22	0.34911 (12)	0.97828 (10)	1.04684 (7)	0.0270 (3)	
H22A	0.3742	0.9917	1.0986	0.032*	
C23	0.41963 (11)	0.92322 (9)	1.03119 (7)	0.0231 (2)	
H23A	0.4914	0.8994	1.0724	0.028*	
C24	0.38282 (10)	0.90363 (8)	0.95362 (6)	0.0178 (2)	
C25	0.18339 (9)	0.79109 (7)	0.70891 (6)	0.01322 (18)	
H25A	0.0997	0.8086	0.6791	0.016*	
C26	0.19018 (9)	0.68381 (8)	0.69502 (6)	0.01475 (19)	
C27	0.08894 (10)	0.63165 (9)	0.64255 (7)	0.0196 (2)	
H27A	0.0161	0.6625	0.6176	0.024*	
C28	0.09463 (12)	0.53367 (9)	0.62668 (8)	0.0268 (3)	
H28A	0.0263	0.4998	0.5913	0.032*	
C29	0.20283 (12)	0.48772 (9)	0.66404 (9)	0.0277 (3)	
H29A	0.2067	0.4223	0.6541	0.033*	
C30	0.30598 (11)	0.53789 (9)	0.71632 (8)	0.0229 (2)	
H30A	0.3785	0.5066	0.7409	0.028*	
C31	0.29907 (10)	0.63545 (8)	0.73123 (7)	0.0170 (2)	
C32	0.45303 (13)	1.28244 (10)	0.82399 (9)	0.0301 (3)	
H32A	0.5239	1.2655	0.8268	0.045*	
H32B	0.4723	1.3029	0.8749	0.045*	
H32C	0.4140	1.3344	0.7870	0.045*	
C33	0.18282 (14)	0.74382 (13)	0.46205 (9)	0.0394 (4)	
H33A	0.2638	0.7465	0.4782	0.059*	
H33B	0.1331	0.7559	0.4066	0.059*	
H33C	0.1657	0.6807	0.4734	0.059*	
C34	0.55698 (11)	0.81167 (11)	0.99437 (8)	0.0296 (3)	
H34A	0.5957	0.7803	0.9723	0.044*	
H34B	0.5423	0.7651	1.0232	0.044*	
H34C	0.6063	0.8627	1.0291	0.044*	
C35	0.50914 (11)	0.64911 (11)	0.81418 (9)	0.0313 (3)	
H35A	0.5685	0.6981	0.8415	0.047*	
H35B	0.5156	0.6215	0.7733	0.047*	
H35C	0.5198	0.5990	0.8504	0.047*	
O6	0.5000	0.5000	1.0000	0.1179 (14)	
C36A	0.4158 (5)	0.5316 (5)	0.9475 (3)	0.0687 (15)	0.50
H36A	0.4078	0.5047	0.9006	0.082*	0.50
H36B	0.4287	0.6008	0.9469	0.082*	0.50
C37A	0.2995 (6)	0.5198 (5)	0.9382 (5)	0.0812 (17)	0.50
H37A	0.2391	0.5488	0.8895	0.122*	0.50
H37B	0.3015	0.5511	0.9811	0.122*	0.50
H37C	0.2830	0.4519	0.9379	0.122*	0.50
C38A	0.6159 (5)	0.4889 (4)	1.0238 (3)	0.0600 (12)	0.50
H38A	0.6444	0.5537	1.0242	0.072*	0.50
H38B	0.6143	0.4541	0.9817	0.072*	0.50
C39A	0.7123 (6)	0.4408 (4)	1.1000 (3)	0.0737 (18)	0.50
H39A	0.7854	0.4426	1.1028	0.111*	0.50
H39B	0.6912	0.3745	1.1009	0.111*	0.50
H39C	0.7210	0.4751	1.1440	0.111*	0.50

### Atomic displacement parameters (Å^2^)

**Table:**

	*U*^11^	*U*^22^	*U*^33^	*U*^12^	*U*^13^	*U*^23^
O1	0.0177 (4)	0.0172 (4)	0.0257 (4)	−0.0036 (3)	0.0114 (3)	−0.0021 (3)
O2	0.0307 (5)	0.0253 (5)	0.0222 (4)	0.0058 (4)	0.0168 (4)	−0.0031 (3)
O3	0.0154 (4)	0.0208 (4)	0.0313 (5)	−0.0015 (3)	0.0144 (3)	−0.0044 (3)
O4	0.0152 (3)	0.0229 (4)	0.0167 (4)	0.0030 (3)	0.0036 (3)	0.0012 (3)
O5	0.0131 (3)	0.0185 (4)	0.0276 (4)	0.0024 (3)	0.0073 (3)	0.0005 (3)
N1	0.0140 (4)	0.0125 (4)	0.0120 (4)	0.0023 (3)	0.0052 (3)	−0.0002 (3)
N2	0.0150 (4)	0.0122 (4)	0.0127 (4)	−0.0003 (3)	0.0059 (3)	0.0000 (3)
C1	0.0188 (5)	0.0149 (5)	0.0157 (4)	0.0005 (4)	0.0094 (4)	0.0009 (4)
C2	0.0313 (6)	0.0156 (5)	0.0242 (6)	0.0004 (4)	0.0150 (5)	−0.0037 (4)
C3	0.0357 (7)	0.0227 (6)	0.0273 (6)	0.0084 (5)	0.0197 (5)	−0.0024 (5)
C4	0.0234 (5)	0.0294 (6)	0.0212 (5)	0.0096 (5)	0.0141 (5)	0.0017 (5)
C5	0.0171 (5)	0.0212 (5)	0.0170 (5)	0.0021 (4)	0.0098 (4)	0.0003 (4)
C6	0.0167 (4)	0.0132 (4)	0.0127 (4)	0.0024 (3)	0.0081 (4)	0.0016 (3)
C7	0.0129 (4)	0.0120 (4)	0.0134 (4)	0.0005 (3)	0.0072 (3)	0.0000 (3)
C8	0.0107 (4)	0.0129 (4)	0.0130 (4)	−0.0001 (3)	0.0054 (3)	−0.0002 (3)
C9	0.0136 (4)	0.0123 (4)	0.0161 (4)	0.0006 (3)	0.0082 (4)	0.0018 (3)
C10	0.0128 (4)	0.0125 (4)	0.0151 (4)	0.0010 (3)	0.0080 (3)	0.0004 (3)
C11	0.0156 (4)	0.0134 (4)	0.0134 (4)	0.0013 (3)	0.0086 (4)	0.0007 (3)
C12	0.0184 (5)	0.0136 (4)	0.0113 (4)	0.0010 (4)	0.0062 (4)	0.0008 (3)
C13	0.0180 (5)	0.0192 (5)	0.0152 (5)	−0.0005 (4)	0.0068 (4)	−0.0007 (4)
C14	0.0198 (5)	0.0243 (6)	0.0206 (5)	−0.0034 (4)	0.0053 (4)	−0.0008 (4)
C15	0.0277 (6)	0.0200 (6)	0.0187 (5)	−0.0036 (5)	0.0029 (5)	−0.0023 (4)
C16	0.0345 (6)	0.0159 (5)	0.0147 (5)	0.0025 (4)	0.0076 (5)	−0.0017 (4)
C17	0.0262 (5)	0.0149 (5)	0.0137 (4)	0.0041 (4)	0.0095 (4)	0.0014 (4)
C18	0.0126 (4)	0.0134 (4)	0.0125 (4)	−0.0004 (3)	0.0062 (3)	0.0004 (3)
C19	0.0163 (4)	0.0158 (5)	0.0132 (4)	−0.0037 (4)	0.0076 (4)	−0.0010 (3)
C20	0.0223 (5)	0.0203 (5)	0.0186 (5)	−0.0031 (4)	0.0123 (4)	−0.0020 (4)
C21	0.0317 (6)	0.0284 (6)	0.0218 (6)	−0.0049 (5)	0.0180 (5)	−0.0063 (5)
C22	0.0347 (7)	0.0304 (7)	0.0155 (5)	−0.0101 (5)	0.0133 (5)	−0.0054 (5)
C23	0.0243 (5)	0.0235 (6)	0.0142 (5)	−0.0074 (4)	0.0057 (4)	0.0006 (4)
C24	0.0180 (5)	0.0167 (5)	0.0150 (5)	−0.0038 (4)	0.0067 (4)	0.0001 (4)
C25	0.0127 (4)	0.0124 (4)	0.0139 (4)	0.0001 (3)	0.0069 (3)	−0.0004 (3)
C26	0.0155 (4)	0.0124 (4)	0.0170 (4)	−0.0006 (3)	0.0093 (4)	−0.0003 (3)
C27	0.0178 (5)	0.0179 (5)	0.0233 (5)	−0.0029 (4)	0.0113 (4)	−0.0051 (4)
C28	0.0269 (6)	0.0198 (6)	0.0349 (7)	−0.0075 (5)	0.0177 (5)	−0.0108 (5)
C29	0.0351 (7)	0.0133 (5)	0.0418 (7)	−0.0025 (5)	0.0257 (6)	−0.0055 (5)
C30	0.0259 (6)	0.0151 (5)	0.0320 (6)	0.0046 (4)	0.0185 (5)	0.0030 (4)
C31	0.0172 (5)	0.0142 (5)	0.0207 (5)	0.0007 (4)	0.0110 (4)	0.0021 (4)
C32	0.0307 (6)	0.0221 (6)	0.0381 (7)	−0.0109 (5)	0.0192 (6)	−0.0022 (5)
C33	0.0403 (8)	0.0449 (9)	0.0263 (7)	0.0213 (7)	0.0140 (6)	−0.0079 (6)
C34	0.0184 (5)	0.0324 (7)	0.0247 (6)	0.0049 (5)	0.0032 (5)	0.0074 (5)
C35	0.0154 (5)	0.0327 (7)	0.0389 (7)	0.0075 (5)	0.0104 (5)	0.0038 (6)
O6	0.109 (3)	0.102 (3)	0.186 (5)	−0.011 (2)	0.108 (3)	−0.055 (3)
C36A	0.078 (4)	0.080 (4)	0.063 (3)	−0.039 (3)	0.049 (3)	−0.018 (3)
C37A	0.088 (4)	0.072 (4)	0.110 (5)	−0.019 (3)	0.071 (4)	−0.032 (4)
C38A	0.059 (3)	0.078 (3)	0.048 (2)	−0.018 (3)	0.033 (2)	−0.005 (2)
C39A	0.125 (5)	0.053 (3)	0.076 (3)	0.027 (3)	0.076 (4)	0.023 (3)

### Geometric parameters (Å, °)

**Table:**

O1—C1	1.3737 (14)	C21—H21A	0.9300
O1—C32	1.4258 (15)	C22—C23	1.387 (2)
O2—C17	1.3654 (16)	C22—H22A	0.9300
O2—C33	1.4281 (16)	C23—C24	1.3952 (16)
O3—C9	1.2186 (13)	C23—H23A	0.9300
O4—C24	1.3701 (15)	C25—C26	1.5156 (15)
O4—C34	1.4281 (14)	C25—H25A	0.9800
O5—C31	1.3723 (14)	C26—C27	1.3916 (15)
O5—C35	1.4243 (14)	C26—C31	1.4061 (15)
N1—C7	1.4668 (13)	C27—C28	1.3984 (17)
N1—C11	1.4669 (14)	C27—H27A	0.9300
N1—H1	0.8713	C28—C29	1.3825 (19)
N2—C25	1.4748 (13)	C28—H28A	0.9300
N2—C18	1.4757 (14)	C29—C30	1.3919 (19)
N2—H2	0.8733	C29—H29A	0.9300
C1—C2	1.3929 (16)	C30—C31	1.3909 (16)
C1—C6	1.4056 (15)	C30—H30A	0.9300
C2—C3	1.393 (2)	C32—H32A	0.9600
C2—H2A	0.9300	C32—H32B	0.9600
C3—C4	1.381 (2)	C32—H32C	0.9600
C3—H3A	0.9300	C33—H33A	0.9600
C4—C5	1.3948 (17)	C33—H33B	0.9600
C4—H4A	0.9300	C33—H33C	0.9600
C5—C6	1.3914 (15)	C34—H34A	0.9600
C5—H5A	0.9300	C34—H34B	0.9600
C6—C7	1.5122 (15)	C34—H34C	0.9600
C7—C8	1.5637 (14)	C35—H35A	0.9600
C7—H7A	0.9800	C35—H35B	0.9600
C8—C9	1.5036 (15)	C35—H35C	0.9600
C8—C18	1.5676 (15)	O6—C36A^i^	1.140 (7)
C8—H8A	0.9800	O6—C36A	1.140 (6)
C9—C10	1.5075 (14)	O6—C38A	1.378 (6)
C10—C11	1.5662 (14)	O6—C38A^i^	1.378 (6)
C10—C25	1.5713 (15)	C36A—C38A^i^	0.939 (6)
C10—H10A	0.9800	C36A—C37A	1.492 (8)
C11—C12	1.5181 (15)	C36A—C39A^i^	1.504 (8)
C11—H11A	0.9800	C36A—H36A	0.9700
C12—C13	1.3905 (16)	C36A—H36B	0.9700
C12—C17	1.4110 (16)	C37A—C39A^i^	0.885 (8)
C13—C14	1.3943 (16)	C37A—C38A^i^	0.979 (7)
C13—H13A	0.9300	C37A—H37A	0.9600
C14—C15	1.387 (2)	C37A—H37B	0.9600
C14—H14A	0.9300	C37A—H37C	0.9600
C15—C16	1.389 (2)	C38A—C36A^i^	0.939 (6)
C15—H15A	0.9300	C38A—C37A^i^	0.979 (8)
C16—C17	1.3947 (17)	C38A—C39A	1.527 (8)
C16—H16A	0.9300	C38A—H38A	0.9700
C18—C19	1.5143 (15)	C38A—H38B	0.9700
C18—H18A	0.9800	C39A—C37A^i^	0.885 (8)
C19—C20	1.3931 (17)	C39A—C36A^i^	1.504 (8)
C19—C24	1.4070 (15)	C39A—H39A	0.9600
C20—C21	1.3985 (17)	C39A—H39B	0.9600
C20—H20A	0.9300	C39A—H39C	0.9600
C21—C22	1.385 (2)		
			
C1—O1—C32	118.28 (10)	C26—C25—H25A	106.9
C17—O2—C33	117.96 (11)	C10—C25—H25A	106.9
C24—O4—C34	117.55 (10)	C27—C26—C31	118.07 (10)
C31—O5—C35	117.90 (10)	C27—C26—C25	120.69 (10)
C7—N1—C11	113.09 (8)	C31—C26—C25	121.14 (9)
C7—N1—H1	108.6	C26—C27—C28	121.23 (11)
C11—N1—H1	108.8	C26—C27—H27A	119.4
C25—N2—C18	110.91 (8)	C28—C27—H27A	119.4
C25—N2—H2	111.8	C29—C28—C27	119.37 (11)
C18—N2—H2	108.2	C29—C28—H28A	120.3
O1—C1—C2	123.61 (10)	C27—C28—H28A	120.3
O1—C1—C6	115.51 (10)	C28—C29—C30	120.93 (11)
C2—C1—C6	120.88 (11)	C28—C29—H29A	119.5
C1—C2—C3	119.30 (12)	C30—C29—H29A	119.5
C1—C2—H2A	120.4	C31—C30—C29	119.08 (11)
C3—C2—H2A	120.4	C31—C30—H30A	120.5
C4—C3—C2	120.79 (12)	C29—C30—H30A	120.5
C4—C3—H3A	119.6	O5—C31—C30	123.54 (10)
C2—C3—H3A	119.6	O5—C31—C26	115.16 (10)
C3—C4—C5	119.46 (12)	C30—C31—C26	121.30 (10)
C3—C4—H4A	120.3	O1—C32—H32A	109.5
C5—C4—H4A	120.3	O1—C32—H32B	109.5
C6—C5—C4	121.27 (11)	H32A—C32—H32B	109.5
C6—C5—H5A	119.4	O1—C32—H32C	109.5
C4—C5—H5A	119.4	H32A—C32—H32C	109.5
C5—C6—C1	118.28 (10)	H32B—C32—H32C	109.5
C5—C6—C7	122.83 (10)	O2—C33—H33A	109.5
C1—C6—C7	118.80 (10)	O2—C33—H33B	109.5
N1—C7—C6	111.03 (8)	H33A—C33—H33B	109.5
N1—C7—C8	108.04 (8)	O2—C33—H33C	109.5
C6—C7—C8	112.91 (8)	H33A—C33—H33C	109.5
N1—C7—H7A	108.2	H33B—C33—H33C	109.5
C6—C7—H7A	108.2	O4—C34—H34A	109.5
C8—C7—H7A	108.2	O4—C34—H34B	109.5
C9—C8—C7	102.19 (8)	H34A—C34—H34B	109.5
C9—C8—C18	111.09 (8)	O4—C34—H34C	109.5
C7—C8—C18	112.34 (8)	H34A—C34—H34C	109.5
C9—C8—H8A	110.3	H34B—C34—H34C	109.5
C7—C8—H8A	110.3	O5—C35—H35A	109.5
C18—C8—H8A	110.3	O5—C35—H35B	109.5
O3—C9—C8	123.61 (10)	H35A—C35—H35B	109.5
O3—C9—C10	124.45 (10)	O5—C35—H35C	109.5
C8—C9—C10	111.46 (9)	H35A—C35—H35C	109.5
C9—C10—C11	105.99 (8)	H35B—C35—H35C	109.5
C9—C10—C25	109.83 (8)	C36A^i^—O6—C36A	179.998 (4)
C11—C10—C25	112.32 (8)	C36A—O6—C38A	137.5 (3)
C9—C10—H10A	109.5	C36A^i^—O6—C38A^i^	137.5 (3)
C11—C10—H10A	109.5	C38A—O6—C38A^i^	179.998 (2)
C25—C10—H10A	109.5	C38A^i^—C36A—O6	82.4 (6)
N1—C11—C12	110.07 (9)	O6—C36A—C37A	121.7 (6)
N1—C11—C10	109.79 (8)	C38A^i^—C36A—C39A^i^	73.3 (5)
C12—C11—C10	110.10 (8)	O6—C36A—C39A^i^	155.6 (5)
N1—C11—H11A	109.0	C38A^i^—C36A—H36A	130.7
C12—C11—H11A	109.0	O6—C36A—H36A	106.9
C10—C11—H11A	109.0	C37A—C36A—H36A	106.9
C13—C12—C17	118.28 (10)	C39A^i^—C36A—H36A	89.9
C13—C12—C11	122.40 (10)	C38A^i^—C36A—H36B	116.7
C17—C12—C11	119.23 (10)	O6—C36A—H36B	106.9
C12—C13—C14	121.41 (11)	C37A—C36A—H36B	106.9
C12—C13—H13A	119.3	C39A^i^—C36A—H36B	84.1
C14—C13—H13A	119.3	H36A—C36A—H36B	106.7
C15—C14—C13	119.40 (13)	C39A^i^—C37A—C38A^i^	109.9 (9)
C15—C14—H14A	120.3	C39A^i^—C37A—C36A	73.5 (7)
C13—C14—H14A	120.3	C38A^i^—C37A—H37A	146.8
C14—C15—C16	120.65 (11)	C36A—C37A—H37A	109.5
C14—C15—H15A	119.7	C39A^i^—C37A—H37B	115.0
C16—C15—H15A	119.7	C38A^i^—C37A—H37B	84.2
C15—C16—C17	119.66 (12)	C36A—C37A—H37B	109.5
C15—C16—H16A	120.2	H37A—C37A—H37B	109.5
C17—C16—H16A	120.2	C39A^i^—C37A—H37C	131.3
O2—C17—C16	124.34 (11)	C38A^i^—C37A—H37C	93.1
O2—C17—C12	115.09 (10)	C36A—C37A—H37C	109.5
C16—C17—C12	120.57 (12)	H37A—C37A—H37C	109.5
N2—C18—C19	111.18 (8)	H37B—C37A—H37C	109.5
N2—C18—C8	112.77 (9)	C36A^i^—C38A—C37A^i^	102.1 (8)
C19—C18—C8	111.73 (8)	C36A^i^—C38A—O6	55.1 (5)
N2—C18—H18A	106.9	C37A^i^—C38A—O6	155.5 (7)
C19—C18—H18A	106.9	C36A^i^—C38A—C39A	70.6 (5)
C8—C18—H18A	106.9	O6—C38A—C39A	125.7 (4)
C20—C19—C24	118.01 (10)	C36A^i^—C38A—H38A	125.9
C20—C19—C18	120.45 (10)	C37A^i^—C38A—H38A	79.5
C24—C19—C18	121.53 (10)	O6—C38A—H38A	105.9
C19—C20—C21	121.53 (11)	C39A—C38A—H38A	105.9
C19—C20—H20A	119.2	C36A^i^—C38A—H38B	127.1
C21—C20—H20A	119.2	C37A^i^—C38A—H38B	95.0
C22—C21—C20	119.34 (12)	O6—C38A—H38B	105.9
C22—C21—H21A	120.3	C39A—C38A—H38B	105.9
C20—C21—H21A	120.3	H38A—C38A—H38B	106.2
C21—C22—C23	120.48 (11)	C37A^i^—C39A—C36A^i^	72.1 (7)
C21—C22—H22A	119.8	C37A^i^—C39A—H39A	74.0
C23—C22—H22A	119.8	C36A^i^—C39A—H39A	145.6
C22—C23—C24	119.95 (11)	C38A—C39A—H39A	109.5
C22—C23—H23A	120.0	C37A^i^—C39A—H39B	133.4
C24—C23—H23A	120.0	C36A^i^—C39A—H39B	90.0
O4—C24—C23	123.94 (10)	C38A—C39A—H39B	109.5
O4—C24—C19	115.38 (10)	H39A—C39A—H39B	109.5
C23—C24—C19	120.68 (11)	C37A^i^—C39A—H39C	112.7
N2—C25—C26	111.42 (8)	C36A^i^—C39A—H39C	89.1
N2—C25—C10	113.87 (8)	C38A—C39A—H39C	109.5
C26—C25—C10	110.31 (9)	H39A—C39A—H39C	109.5
N2—C25—H25A	106.9	H39B—C39A—H39C	109.5
			
C32—O1—C1—C2	−13.03 (17)	C7—C8—C18—C19	−114.20 (10)
C32—O1—C1—C6	166.55 (11)	N2—C18—C19—C20	−125.03 (11)
O1—C1—C2—C3	−179.41 (11)	C8—C18—C19—C20	108.00 (11)
C6—C1—C2—C3	1.03 (18)	N2—C18—C19—C24	55.84 (13)
C1—C2—C3—C4	0.4 (2)	C8—C18—C19—C24	−71.13 (13)
C2—C3—C4—C5	−0.8 (2)	C24—C19—C20—C21	−0.32 (17)
C3—C4—C5—C6	−0.13 (18)	C18—C19—C20—C21	−179.47 (11)
C4—C5—C6—C1	1.47 (16)	C19—C20—C21—C22	−0.40 (19)
C4—C5—C6—C7	−174.94 (10)	C20—C21—C22—C23	0.3 (2)
O1—C1—C6—C5	178.48 (10)	C21—C22—C23—C24	0.59 (19)
C2—C1—C6—C5	−1.92 (16)	C34—O4—C24—C23	3.27 (17)
O1—C1—C6—C7	−4.96 (14)	C34—O4—C24—C19	−176.92 (10)
C2—C1—C6—C7	174.64 (10)	C22—C23—C24—O4	178.47 (11)
C11—N1—C7—C6	172.53 (9)	C22—C23—C24—C19	−1.33 (18)
C11—N1—C7—C8	−63.14 (11)	C20—C19—C24—O4	−178.64 (10)
C5—C6—C7—N1	19.29 (14)	C18—C19—C24—O4	0.51 (15)
C1—C6—C7—N1	−157.11 (9)	C20—C19—C24—C23	1.18 (16)
C5—C6—C7—C8	−102.23 (11)	C18—C19—C24—C23	−179.67 (10)
C1—C6—C7—C8	81.38 (12)	C18—N2—C25—C26	175.83 (9)
N1—C7—C8—C9	64.88 (10)	C18—N2—C25—C10	50.28 (11)
C6—C7—C8—C9	−171.93 (9)	C9—C10—C25—N2	5.95 (12)
N1—C7—C8—C18	−54.25 (11)	C11—C10—C25—N2	−111.74 (9)
C6—C7—C8—C18	68.94 (11)	C9—C10—C25—C26	−120.19 (9)
C7—C8—C9—O3	103.39 (11)	C11—C10—C25—C26	122.13 (9)
C18—C8—C9—O3	−136.60 (11)	N2—C25—C26—C27	121.40 (11)
C7—C8—C9—C10	−68.94 (10)	C10—C25—C26—C27	−111.10 (11)
C18—C8—C9—C10	51.06 (11)	N2—C25—C26—C31	−62.18 (14)
O3—C9—C10—C11	−107.83 (12)	C10—C25—C26—C31	65.32 (13)
C8—C9—C10—C11	64.43 (11)	C31—C26—C27—C28	0.68 (18)
O3—C9—C10—C25	130.62 (11)	C25—C26—C27—C28	177.21 (12)
C8—C9—C10—C25	−57.13 (11)	C26—C27—C28—C29	0.2 (2)
C7—N1—C11—C12	178.51 (8)	C27—C28—C29—C30	−0.8 (2)
C7—N1—C11—C10	57.16 (11)	C28—C29—C30—C31	0.5 (2)
C9—C10—C11—N1	−54.43 (11)	C35—O5—C31—C30	4.10 (18)
C25—C10—C11—N1	65.51 (11)	C35—O5—C31—C26	−175.12 (11)
C9—C10—C11—C12	−175.76 (9)	C29—C30—C31—O5	−178.70 (12)
C25—C10—C11—C12	−55.83 (11)	C29—C30—C31—C26	0.47 (19)
N1—C11—C12—C13	−13.88 (14)	C27—C26—C31—O5	178.20 (10)
C10—C11—C12—C13	107.29 (12)	C25—C26—C31—O5	1.69 (16)
N1—C11—C12—C17	169.67 (9)	C27—C26—C31—C30	−1.03 (17)
C10—C11—C12—C17	−69.17 (13)	C25—C26—C31—C30	−177.55 (11)
C17—C12—C13—C14	0.85 (17)	C38A—O6—C36A—C38A^i^	180.000 (7)
C11—C12—C13—C14	−175.64 (11)	C38A—O6—C36A—C37A	172.7 (5)
C12—C13—C14—C15	0.52 (18)	C38A^i^—O6—C36A—C37A	−7.3 (5)
C13—C14—C15—C16	−0.91 (19)	C38A—O6—C36A—C39A^i^	−178.6 (11)
C14—C15—C16—C17	−0.10 (19)	C38A^i^—O6—C36A—C39A^i^	1.4 (11)
C33—O2—C17—C16	−11.08 (17)	C38A^i^—C36A—C37A—C39A^i^	162.4 (11)
C33—O2—C17—C12	169.62 (11)	O6—C36A—C37A—C39A^i^	173.7 (7)
C15—C16—C17—O2	−177.76 (11)	O6—C36A—C37A—C38A^i^	11.3 (7)
C15—C16—C17—C12	1.51 (17)	C39A^i^—C36A—C37A—C38A^i^	−162.4 (11)
C13—C12—C17—O2	177.47 (10)	C36A—O6—C38A—C36A^i^	179.999 (10)
C11—C12—C17—O2	−5.94 (15)	C36A^i^—O6—C38A—C37A^i^	−23.4 (15)
C13—C12—C17—C16	−1.86 (16)	C36A—O6—C38A—C37A^i^	156.6 (15)
C11—C12—C17—C16	174.74 (10)	C36A^i^—O6—C38A—C39A	0.7 (5)
C25—N2—C18—C19	176.88 (8)	C36A—O6—C38A—C39A	−179.3 (5)
C25—N2—C18—C8	−56.72 (11)	C36A^i^—C38A—C39A—C37A^i^	−161.3 (11)
C9—C8—C18—N2	5.92 (11)	O6—C38A—C39A—C37A^i^	−161.9 (10)
C7—C8—C18—N2	119.70 (9)	C37A^i^—C38A—C39A—C36A^i^	161.3 (11)
C9—C8—C18—C19	132.02 (9)	O6—C38A—C39A—C36A^i^	−0.6 (5)

Symmetry codes: (i) −*x*+1, −*y*+1, −*z*+2.

### Hydrogen-bond geometry (Å, °)

**Table:**

*D*—H···*A*	*D*—H	H···*A*	*D*···*A*	*D*—H···*A*
N2—H2···O4	0.87	2.19	2.8184 (13)	129
N2—H2···O5	0.87	2.32	2.8936 (15)	124
C4—H4A···N2^ii^	0.93	2.60	3.470 (2)	156
N1—H1···Cg1^iii^	0.87	2.66	3.4167 (10)	146
C34—H34C···Cg2^iv^	0.96	2.70	3.5099 (16)	143

Symmetry codes: (ii) −*x*, *y*+1/2, −*z*+3/2; (iii) −*x*, −*y*+2, −*z*+1; (iv) −*x*+1, −*y*+2, −*z*+2.
